# n-Propanol extract of boiled and fermented koro benguk (*Mucuna pruriens* seed) shows a neuroprotective effect in paraquat dichloride-induced Parkinson’s disease rat model

**DOI:** 10.14202/vetworld.2018.1250-1254

**Published:** 2018-09-12

**Authors:** Yosua Kristian Adi, Rini Widayanti, Tri Wahyu Pangestiningsih

**Affiliations:** 1 Master Program of Veterinary Science, Faculty of Veterinary Medicine, Universitas Gadjah Mada, Yogyakarta, Indonesia; 2 Department of Biochemistry, Faculty of Veterinary Medicine, Universitas Gadjah Mada, Yogyakarta, Indonesia; 3 Department of Anatomy, Faculty of Veterinary Medicine, Universitas Gadjah Mada, Yogyakarta, Indonesia

**Keywords:** animal model, *Mucuna pruriens*, neuroprotective, paraquat dichloride, Parkinson’s disease

## Abstract

**Aim:**

n-Propanol extracts from fresh, boiled, and fermented seeds were studied to evaluate their neuroprotective effects in a Parkinson’s disease (PD) rat model, based on the total number of dopaminergic (DA) neurons in the substantia nigra pars compacta (SNpc).

**Materials and Methods:**

Rats were induced with paraquat dichloride at a dosage of 7 mg/kg BW intraperitoneally twice a week and at the same time supplemented with extract at a dosage of 70 mg/kg BW orally every day for 3 weeks. On the 24^th^ day, all rats were perfused and fixed with 4% paraformaldehyde. The left part of the SNpc was processed for immunohistochemical staining with tyrosine hydroxylase (TH)-antibody. The total number of DA neurons in SNpc was evaluated with a stereological method.

**Results:**

TH-immunoreactive cells found in the SNpc were identified as DA neurons. The average total number of DA neurons in the SNpc increased significantly in the PD rat model that was given an n-propanol extract of boiled and fermented seeds compared with a control PD rat model. Surprisingly, there was no significant difference in the average total number of DA neurons in SNpc between the PD rat model that was given n-propanol extract of fresh seeds and the control PD rat model.

**Conclusion:**

n-Propanol extract of boiled and fermented seeds could produce a higher neuroprotective effect against DA neuron than fresh seeds in a PD rat model.

## Introduction

At present, human life expectancy is increasing. Based on data from the World Health Organization, the number of people aged over 65 will have risen from 390 million in 1997 to 800 million in 2025 [1]. However, aging of the population is also followed by an increasing number of patients with degenerative diseases. Mortality from such neurodegenerative diseases as Parkinson’s disease (PD), Alzheimer’s, and cerebrovascular disease will increase, contrary to that of infectious diseases such as tetanus, meningitis, and Japanese encephalitis [2]. PD was first reported by James Parkinson in 1817 in London, but the concept of the disease continued to evolve the present level of understanding. PD is characterized by loss of dopaminergic (DA) neurons in the substantia nigra pars compacta (SNpc) and the presence of intraneuronal inclusions (Lewy bodies) [3]. It has multifactorial causes such as infection at a young age, environmental factors, neurotoxic substances (paraquat [PQ] dichloride, rotenone, maneb, MPTP, etc.), metabolic diseases, neurodegenerative diseases, and genetic factors [3-5]. To generate disease symptoms, the loss of nigral neurons must be at least 50%, and at autopsy, most cases show a greater than 80% reduction [6]. The loss of nigral neurons will result in a deficiency of dopamine within the basal ganglia, leading to a movement disorder known as parkinsonian symptoms. These include bradykinesia, muscular rigidity, rest tremor, and postural, and gait impairment [7].

To date, available therapies for PD only treat symptoms of the disease. Several drugs that enhance intracerebral dopamine concentrations or stimulates dopamine receptors include levodopa (L-DOPA), dopamine agonists, and monoamine-oxidase inhibitors; these are effective at alleviating PD symptoms [7,8]. L-DOPA has proven to be the most reliable and efficacious for the restoration of dopamine in PD. Unlike dopamine, L-DOPA is transported through the blood-brain barrier by the L-system large amino neutral amino acid transporter. Once it transported to the central nervous system, L-DOPA is converted into dopamine by aromatic-amino-acid decarboxylase [8]. Since it can be found in *Mucuna pruriens* (MP) seeds, studies aiming to evaluate the effect of MP supplementation in PD patients have been held in many countries. However, the pharmaceutical composition of MP seeds, the use of MP seed powder, and the method of extracting the substance from MP have been reported by Giessen *et al*. [9] in a United States Patent Application Publication. In Indonesia, particularly in the Yogyakarta region and surrounding areas, MP seed is known as koro benguk. It has been utilized as the main ingredients to made “tempe koro benguk,” a product of fermented MP seeds. In addition, L-DOPA can still be extracted from this form.

The neuroprotective effects of MP extract in a PD animal model have been reported by many researchers [10-14]. However, the neuroprotective effect of n-propanol extract from tempe koro benguk and boiled koro benguk has not been studied. This research was carried out to reveal the neuroprotective effect of n-propanol extract of boiled and fermented koro benguk, as an alternative drug for PD prevention in the future.

## Materials and Methods

### Ethical approval

All the methods and treatments of animals were approved by the Ethical Clearance Committee of Integrated Research and Examination Laboratory (LPPT), Universitas Gadjah Mada, with the certificate number: 00018/04/LPPT/V/2016.

### Experimental animals

25 male adult Wistar rats were used in this research. Male rats were selected to homogenize the experimental animals since female rats have hormonal cycles that can affect the experiment. These rats were divided into five groups. Group I (n=5), as the healthy control group, received an injection of Aqua Pro Injection (DW-14-R5, Aqua pro Injection, PT. IKAPHARMINDO PUTRAMAS, Jakarta-Indonesia) 1 ml/kg BW intraperitoneally twice a week for 3 weeks. Group II (n=5), as the PD-induced group, was injected with PQ dichloride at a dose of 7 mg/kg BW intraperitoneally twice a week for 3 weeks. Injection of PQ dichloride intraperitoneally with appropriate dose and period will lead parkinsonism in rats. The other fifteen rats were divided into three groups, IIIA (n=5), IIIB (n=5), and IIIC (n=5). These groups received PQ induction and extracted supplementation in the same week. The PQ induction protocol was the same as in Group II, and the extract administered to Group IIIA, IIIB, and IIIC, respectively, were fresh koro benguk n-propanol extract, boiled koro benguk n-propanol extract, and fermented koro benguk n-propanol extract. The dose of extract for all three groups was 70 mg/kg BW, given orally every day for 3 weeks. Koro benguk seeds were obtained from Kulon Progo, Special Administrative Region of Yogyakarta, Indonesia. Koro benguk plant was identified in Laboratory of Plant Systematics, Faculty of Biology, Universitas Gadjah Mada. The results confirmed that koro benguk we used was the seed of MP. The extractions were done in Integrated Research and Testing Laboratory (LPPT), Universitas Gadjah Mada.

### Sample collection and histological preparation

On day 24^th^, all animals were perfused transcardially with physiological NaCl and fixed with phosphate-buffered paraformaldehyde 4%. The brain was removed then fixed again in the same fixative solution. The SNpc region was determined with the aid of the rat brain atlas that was established by Paxinos and Watson [15]. The left half of the brain was embedded in paraffin then sliced to a thickness of 4 µ to estimate the total number of DA neurons in SNpc based on the stereological method explained by Boyce *et al*. [16]. DA neurons were identified on the basis of immunohistochemical staining with the primary antibody rabbit anti-tyrosine hydroxylase (TH) (1/500, Boster Immunoleader, Cat No: PB9449). The immunoreactivities (IRs) were visualized with the Starr Trek Universal HRP Detection kit (Biocare Medical, Cat No: STUHRP700 H, L10).

### Statistical analysis

Average total numbers of TH-IR neurons in SNpc in all five groups were analyzed by one-way ANOVA using SPSS ver.16 to test for significant differences between the groups.

## Results

Immunovisualization using the Starr Trek Universal HRP Detection system resulted in the formation of a brown precipitate within the cell body and the axonal and dendritic processes. The TH-IR cells that were found in the SNpc, as marked by a brown precipitate within the cell, were identified as DA neurons. Distinct IRs of TH-IR cells were observed between the five groups. We observed a high intensity of TH-IR cells for most of the samples in Group II, in contrast to Group I. The other three groups, in order from the highest to the lowest intensity of IR, were Group IIIC, Group IIIB, and Group IIIA. With the stereology method, the total number of TH-IR cells in the SNpc can be counted without bias. The average total number of TH-IR cells in the SNpc of Group I, the healthy control group, was the highest of all five groups. Group II, as the PD-induced group, showed the lowest average total number of TH-IR cells in the SNpc. Of the three other groups, the highest average total number of TH-IR cells in the SNpc was in Group IIIB, followed by Group IIIC, and finally Group IIIA. The data are shown in Table-1.

**Table-1:** Average total number of TH-IR cells in the SNpc of five groups.

TH-IR cells	3-week treatment (mean±SD)				

	Group I	Group II	Group IIIA	Group IIIB	Group IIIC
Rat 1	22.950	11.424	13.668	21.828	16.218
Rat 2	26.112	13.362	16.218	18.564	21.012
Rat 3	31.416	12.546	14.994	16.626	19.482
Rat 4	25.704	15.606	17.850	19.482	15.810
Rat 5	29.274	10.812	14.688	19.788	18.870
Mean±SD	27.091±3.298	12.750±1.877	15.484±1.605	19.258±1.894	18.278±2.214

SD=Standard deviation, TH-IR=Tyrosine hydroxylase immunoreactive, SNpc=Substantia nigra pars compacta

Statistical analysis revealed significant differences in the average total number of TH-IR cells in the SNpc between the five groups. The total number of TH-IR cells in the SNpc Group II was significantly lower than that in Group I. On the other hand, the total number TH-IR cells in the SNpc in Group IIB and Group IIIC were greater than that in Group II, although they were still lower than that in Group I. Surprisingly, there was no significant difference between Group IIIA and Group II.

## Discussion

Immunohistochemical staining using the primary antibody anti-TH to detect DA neurons has been reported by many researchers [17-19]. The TH-IR found in this study were similar to those reported by Nair-Roberts *et al*. [17], who used the immunolabeling peroxidase-DAB system, which resulted in brown precipitation in the cell body, axon, and dendrite process. Furthermore, Nair-Roberts *et al*. [17] explained that there was also a light brown color in the nucleus of the neuron, although its intensity was always lower than that seen in the cell cytoplasm with a clear visible nucleus. DA neurons could be detected by immunohistochemical staining with primary antibody against dopamine or an enzyme involved in its synthesis [20].

Differences in the intensity of TH-IR in immunohistochemical staining could be interpreted as differences in TH protein expression [21]. The higher intensity of TH-IR cells in the SNpc Group II indicated higher TH protein in DA neurons compared with Group I. The greater need for TH protein to convert tyrosine to L-DOPA before further conversion to dopamine is allegedly caused by the decreasing number of DA neurons in the SNpc of PD-induced rats. TH is “the rate-limiting enzyme” in the catecholamine system [20,22]. When the synapse in catecholamine neurons needs more neurotransmitter, this enzyme would be activated to convert more L-DOPA, since L-DOPA is the precursor for other catecholamine neurotransmitters [22,23]. A higher L-DOPA content in the fresh seed extract compared with the boiled and fermented seed extract [24] reduce the need for TH to convert more L-DOPA in PD-induced rats supplemented with the fresh seed extract. This hypothesis was supported by lower TH-IR in Group IIIA compared with Groups IIIB and IIIC. The TH-IR of Group IIIC was the highest because fermented seeds contained the least L-DOPA.

**Figure-1:**
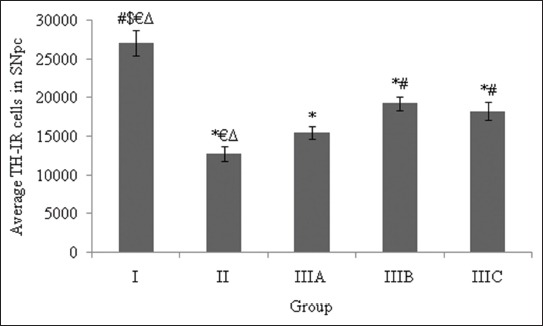
Diagram showing the average total number of TH-IR cells in the SNpc of the five groups. The symbol * indicates a significant difference compared with Group I; # indicates a significant difference compared with Group II; $ indicates a significant difference compared with Group IIIA; € indicates a significant difference compared with Group IIIB; Δ indicates a significant difference compared with Group IIIC (p<0.05).

The data in Figure 1 showed a significant decrease in TH-IR cells in the SNpc of Group II compared with Group I. Induction of PQ at a dosage of 7 mg/kg BW twice a week for 3 weeks decreased the number of DA neurons by approximately 53%. This result was similar to that reported by Somayajulu-Nitu *et al*. [18] that induction of PQ at a dosage of 10 mg/kg BW once a week for 3 weeks could reduce DA neurons in the SNpc about 65%. A different result was offered by Fahim *et al*. [25], who reported a loss of approximately 32% of DA neurons in the SNpc of rats induced with PQ at a dose of 10 mg/kg BW every day for 3 weeks. It seems that the mechanism of DA neuronal death caused by PQ was dose-dependent [26-28]. Toxicity of PQ against cells involves redox cycling and the production of reactive oxygen species (ROS), free radicals that lead to oxidative stress [29,30]. The ROS, especially O_2_^−^ and H_2_O_2_, can cause damage to biological substances, including nucleic acids and amino acids [31]. However, the most destructive effect of free radicals is the induction of lipid peroxidation [32]. The plasma membrane, composed of a phospholipid bilayer, is the primary target of ROS, which causes cell death.

This study revealed evidence that n-propanol extract of processed koro benguk seeds, both boiled and fermented, have a higher neuroprotective effect than fresh seeds. This was shown by the significantly higher average total number of TH-IR cells in Groups IIIB and IIIC compared with Group II, although it could not reach the same level as Group I. In contrast, Group IIIA was not significantly different from Group II as the PD-induced group. This evidence showed that the neuroprotective effect not simply caused by the presence of L-DOPA in koro benguk. The highest L-DOPA content could be found in fresh seed extract [24], but its neuroprotective effect was not as high as that of the extract from boiled and fermented seeds. Yadav *et al*. [14] explained in their report that the neuroprotective effect of koro benguk in a PD animal model comes from an alkaloid antioxidant or another antioxidant such as ursolic acid contained in MP seeds/koro benguk. There are more than 50 substances contained in MP seeds [33]. Which one of the substances and the optimal processing method to use on seeds, that act as the best neuroprotective agents against PD in an animal model requires further exploration.

## Conclusion

The average total number of TH-IR cells in SNpc of a PD rat model administered n-propanol extract of boiled and fermented seeds was statistically significantly higher than in the control PD rat model. There was no significant difference between the average total numbers of TH-IR cells in the SNpc of a PD rat model administered n-propanol extract of fresh seeds and the control PD rat model. n-Propanol extract of boiled and fermented seeds could provide a higher neuroprotective effect against DA neurons than fresh seeds in a PD rat model.

## Authors’ Contributions

TWP designed this study. YKA perform the experimental under the guidance of TWP. YKA and RW analyzed the result. YKA and TWP drafted and revised the manuscript. All the authors read and approved the final manuscript.
